# Proteinaceous determinants of surface colonization in bacteria: bacterial adhesion and biofilm formation from a protein secretion perspective

**DOI:** 10.3389/fmicb.2013.00303

**Published:** 2013-10-14

**Authors:** Caroline Chagnot, Mohamed A. Zorgani, Thierry Astruc, Mickaël Desvaux

**Affiliations:** ^1^UR454 Microbiologie, INRASaint-Genès Champanelle, France; ^2^UR370 Qualité des Produits Animaux, INRASaint-Genès Champanelle, France

**Keywords:** secretome, adhesin, pili/fimbriae/curli, cell surface, aggregation, secreted protein, MSCRAMM, protein secretion system

## Abstract

Bacterial colonization of biotic or abiotic surfaces results from two quite distinct physiological processes, namely bacterial adhesion and biofilm formation. Broadly speaking, a biofilm is defined as the sessile development of microbial cells. Biofilm formation arises following bacterial adhesion but not all single bacterial cells adhering reversibly or irreversibly engage inexorably into a sessile mode of growth. Among molecular determinants promoting bacterial colonization, surface proteins are the most functionally diverse active components. To be present on the bacterial cell surface, though, a protein must be secreted in the first place. Considering the close association of secreted proteins with their cognate secretion systems, the secretome (which refers both to the secretion systems and their protein substrates) is a key concept to apprehend the protein secretion and related physiological functions. The protein secretion systems are here considered in light of the differences in the cell-envelope architecture between diderm-LPS (archetypal Gram-negative), monoderm (archetypal Gram-positive) and diderm-mycolate (archetypal acid-fast) bacteria. Besides, their cognate secreted proteins engaged in the bacterial colonization process are regarded from single protein to supramolecular protein structure as well as the non-classical protein secretion. This state-of-the-art on the complement of the secretome (the secretion systems and their cognate effectors) involved in the surface colonization process in diderm-LPS and monoderm bacteria paves the way for future research directions in the field.

## Introduction

Relative to the bacterial cell, three major classes of interactions can be distinguished: (i) the symbiotic relationships with others biological entities from eukaryotic cells, bacterial cells to viruses (bacteriophages), e.g., mutualism, amensalism, competition, etc…, (ii) the sensing of products or stimuli (cell-cell communication, mechano-physico-chemico sensitivity), and (iii) the direct contacts with surfaces or interfaces. Bacterial colonization corresponds to the presence of microorganisms in a particular environment. Colonization of biotic or abiotic surfaces results from two quite distinct microbiological processes, namely bacterial adhesion or biofilm formation. Initial bacterial adhesion can either be reversible or irreversible. Broadly speaking, a biofilm is defined as the sessile development of microbial cells. The engagement into a sessile mode of growth arises following irreversible bacterial adhesion (Zhao et al., 2013); though, not all single adhered bacterial cells necessarily engage into sessile development. Considering the differences in the molecular physiology of the bacterial cells, the distinction between single adhered and biofilm cells is of importance (Buncic et al., 2013). While initial attachment depends on the physiological state of the cell prior to adhesion, significant changes in gene/protein expression occur upon irreversible adhesion and further sessile cell division to colonize the site of adhesion. In all cases, the bacterial cell envelope plays a critical role in the primary interactions between the bacterial cell and its environment.

Numerous factors promote bacterial cell adhesion to surface and interfaces (An and Friedman, 1998; Shirtliff et al., 2002). They can be categorized into (i) general physico-chemical surface properties, (ii) the exopolymeric matrix, and (iii) the cell surface biochemical components. The physico-chemistry of the interactions (such as van der Waals attraction, gravitational force, electrostatic charge, or hydrophobic interaction) (Gottenbos et al., 2000) have been theorized into models by the thermodynamic (Morra and Cassinelli, 1996) and the DLVO (Derjaguin-Landau-Verwey-Overbeek) theories, as well as its extended version (Jucker et al., 1998; Hermansson, 1999). While those models can help explaining some experimental observations, neither of them can fully describe bacterial adhesion as they fail in taking into account a fundamental properties of a biological system that is its adaptability and variability (Katsikogianni and Missirlis, 2004). Indeed, in the course of adhesion and/or biofilm formation, bacterial cells change their physiology at different regulatory levels, e.g., gene/protein expression. This can induce modifications of cell morphology, general surface properties and/or express specific determinants for adhesion. Among those, the exopolymers can be prominent components synthetized in the course of bacterial adhesion and biofilm formation where they play the role of molecular glue (Flemming and Wingender, 2010). A large variety of exopolymers can be involved in bacterial colonization process namely various exopolysaccharides (EPS), e.g., alginate, cellulose, or poly-*N*-acetylglucosamine (PNAG) (Ryder et al., 2007; Vu et al., 2009; Bazaka et al., 2011), extracellular DNA (eDNA) and polyglutamate (Candela and Fouet, 2006). In some cases, those exoplymers are closely associated with the bacterial cell envelope, such as some polyglutamate covalently linked to peptidoglycan (Candela et al., 2012). Some other components of the cell envelope can participate in biofilm formation such as lipopolysaccharides (LPS) (Nakao et al., 2012), (lipo)teichoic acids (Gross et al., 2001; Fabretti et al., 2006), or mycolic acids (Ojha et al., 2005; Zambrano and Kolter, 2005). Among cell envelope components, proteins are undoubtedly the most functionally diverse active components. To be present on the bacterial cell surface, though, a protein must be secreted in the first place (or released by non-active translocation process, e.g., cell lysis or membrane budding).

Protein secretion is a key event for the presence of effectors at the interface between the bacterial cell and its immediate environment (Henderson et al., 2004). Those effectors can be displayed on the bacterial cell surface following anchoring to the cell envelope, released into the extracellular milieu or even beyond, i.e., within a host cell. Secreted proteins feature the lifestyle of a bacterium, its interaction within the ecosystems, microbiota, and ecological niches; for instance, virulence factors in pathogenic bacteria or degradative enzymes in saprophytic bacteria. As such, protein secretion is a key player in bacterial cell physiology and interactions with their environment. To understand protein secretion systems in bacteria, it is crucial to consider the cell envelope architecture. In recent years, it clearly appeared the grouping of bacteria into Gram-positive and Gram-negative bacteria was not satisfactory but ambiguous to describe and categorize accurately the protein secretion systems. Considering the inherent ambiguities of the Gram-negative and Gram-positive terminology, which can refer to three different and sometimes completely unrelated aspects (i.e., Gram staining, cell envelope architecture, and taxonomic grouping) (Desvaux et al., 2009b), the description of monoderm and diderm bacteria is much more appropriate in the field of protein secretion, at least. Indeed, monoderm bacteria (monodermata) refers specifically to species exhibiting only one biological membrane, i.e., the cytoplasmic membrane (CM), whereas diderm bacteria (didermata) refers specifically to species exhibiting two biological membranes, i.e., a CM, then also called inner membrane (IM) and an outer membrane (OM). Diderm bacteria can be further discriminated into (i) diderm-LPS bacterial cells, which possess an archetypal and assymetrical OM containing lipopolysaccharide (LPS) on the external side, (ii) simple-diderm bacterial cells, which possess an OM lacking LPS, and (iii) diderm-mycolate bacterial cells, where an outer lipid layer resembling an OM is composed of mycolic acid molecules arranged in a highly ordered form (Brennan and Nikaido, 1995; Sutcliffe, 2010; Gupta, 2011).

**Figure 1:**
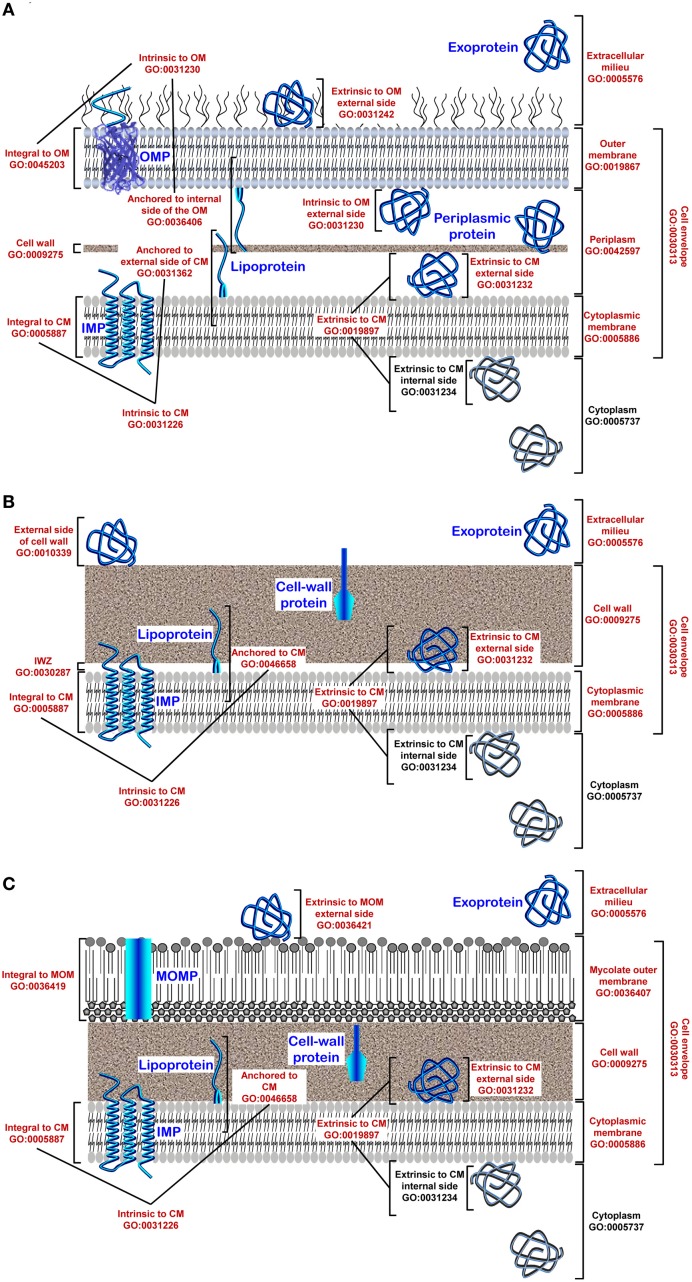
**Gene Ontology (GO) for cellular components and protein subcellular location in bacteria. (A)** In diderm-LPS bacteria, five clearly defined compartments are considered (i) the cytoplasm (CP; GO:0005737), (ii) the cytoplasmic membrane (CM; GO:0005886), (iii) the periplasm (PP; GO:0042597), (iv) the outer membrane (OM; GO:0019867), and (v) the extracellular milieu (EM; GO:0005576). The cell envelope (GO:0010339) is constituted of the OM and CM, also called inner membrane (IM), as well as a thin cell wall (CW) located in-between. The situation at the CM can be discriminated even further between locations either intrinsic (GO:0031226) or extrinsic (GO:0019897) to the CM. The former refers to gene products with covalently attached moieties embedded in the CM, which splits into locations (i) integral to CM (GO:0005887) where some part of the peptide sequence spans all or part of the CM, i.e., the integral membrane proteins (IMPs), and (ii) anchored to the external side of CM (GO: 0031362) corresponding to proteins tethered to the CM by non-polypeptidic covalently attached anchor, i.e., the lipoproteins. The latter refers to proteins extrinsic to the CM, i.e., neither anchored by covalent bonds to any moiety nor directly embedded in the CM. Peripheral proteins are loosely bound to the surface components of the CM on the internal (GO:0031234) or external side (GO:0031232). Some proteins localized at the OM can also be subunits of supramolecular protein complexes (GO:0043234). The situation at the OM can be discriminated even further between locations intrinsic (GO:003230) or extrinsic (GO:0031242) to the OM. The former refers to gene products with a covalently attached moiety embedded in the OM, which splits into locations (i) integral to OM (GO:00045203) where some part of the peptide sequence spans all or part of the OM, i.e., the outer membrane proteins (OMPs), and (ii) anchored to internal side of OM (GO:0036406) corresponding to proteins tethered to the OM by non-polypeptidic covalently attached anchor, i.e., some lipoproteins. **(B)** In monoderm bacteria, four clearly defined compartments are considered (i) the cytoplasm (CP; GO:0005737), (ii) the cytoplasmic membrane (CM; GO:0005886), (iii) the cell wall (CW; GO:0009275), and (iv) the extracellular milieu (EM; GO:0005576). An inner wall zone (IWZ) (Matias and Beveridge, 2005) has been identified (GO:0030287); importantly, it should not be considered *sensu stricto* as a periplasm since the CW is porous and therefore it is not bordered (bounded) contrary to the situation in diderm-LPS bacteria where the periplasmic space is strictly delimited by two biological membranes. The CM and CW constitute the cell envelope (GO:0010339). The situation at the CM is similar to what is described for diderm-LPS bacteria. Some proteins localized at the CM or CW can also be subunits of protein complex (GO:0043234) or be extrinsic to the CW (GO:0010339). **(C)** In diderm-mycolate bacteria, five clearly defined compartments are considered (i) the cytoplasm (CP; GO:0005737), (ii) the cytoplasmic membrane (CM; GO:0005886), (iii) the cell wall (CW; GO:0009275), (iv) the mycolate outer membrane (MOM) or mycomembrane (GO:0036407), and (v) the extracellular milieu (EM; GO:0005576). The cell envelope (GO:0010339) is constituted of the MOM, CW, and CM; a pseudo-periplasm might exist but remains to be evidenced. The situation at the CM is similar to what is described for diderm-LPS or diderm bacteria. The situation at the MOM (GO:0036407) can be discriminated further between location integral to the mycomembrane (GO:0036419), i.e., the MOM proteins (MOMPs), or extrinsic to the MOM (GO:0036420). Location at the cell surface (GO:0009986) refers (i) in diderm-LPS, to the OM and/or external side of the OM and is intended to proteins exposed externally (GO:0031242) or intrinsic to the OM (GO:0031230) (similar rermarks apply to diderm-mycolate bacteria and the MOM), and (ii) in monoderm bacteria, to the CW and/or external side of the CM and is intended to proteins exposed externally or attached to the CW (GO:0009275) or the CM, i.e., integrated (GO:0005887), anchored (GO:0046658) or loosely bound (GO:0031232). As the CW is not a permeability barrier in monoderm bacteria but porous, the surface proteins do not necessarily have domains protruding from the confine of the cell envelope to interact with the external environment. Altogether with the subset of proteins localized extracellularly (GO:0005576), i.e., the exoproteome, these gene products (GO numbers in red) correspond to the extracytoplasmic proteins, i.e., the extracytoproteome (proteins depicted in blue).

Considering the close association of secreted proteins with their cognate secretion systems, the secretome is a key concept to apprehend the protein secretion and related physiological functions. The secretome refers both to the secretion/translocation systems and the protein substrates of these transport systems (Tjalsma et al., 2000; Antelmann et al., 2001, 2006; Van Dijl et al., 2001; Economou, 2002; Sarvas et al., 2004; Buist et al., 2006; Desvaux et al., 2009b). The secretome concept provides an integrated and global view of protein secretion by considering protein routing, transport mechanisms, post-translational modifications, and protein subcellular location. Confusion between the secretome and the secreted/extracellular proteins is a common misunderstanding promulgated in part of the scientific literature but it must be stressed again the secretome is neither the exoproteome (extracellular proteome) nor an “omics” approach *per se* (Desvaux et al., 2009b). The exoproteome refers specifically to the subset of proteins present in the supernatant (which are not necessarily secreted). Secreted proteins are not necessarily free soluble extracellular proteins (exoproteins) since they can have different final subcellular locations (membranes, cell wall, extracellular milieu) or be subunits of supramolecular protein complexes (e.g., flagellum, pilus, cellulosome). Secreted proteins can even have multiple final subcellular locations, which are described following the gene ontology (GO) for “Cellular component” (Figure 1). All extracytoplasmic proteins are not systematically secreted since some exoproteins can be released upon molecular events that are not active translocation process and thus not secretion *per se*, e.g., cell lysis (autolysis, allolysis, bacteriophage lysis), GTA (gene transfer agent) or membrane budding (vesicles). The translocation corresponds to the active transport across a biological membrane (Desvaux et al., 2009b); the secretion refers to active transport from the interior to the exterior of the cell and the export to active transport across the CM (Economou et al., 2006). While in monoderm bacteria secretion and export are synonymous, in diderm bacteria the secretion is completed only upon translocation across the OM. For the sake of clarity, these key definitions in the field of protein secretion are reminded in Table 1.

**Table 1:** **Some key definitions in the field of bacterial protein secretion**.

**Terminology**	**Definition**	**Note^a^**	**References**
Monoderm bacteria	Bacterial species exhibiting only one biological membrane, i.e. the CM.	Corresponds to the archetypal Gram-positive bacteria (i.e., with a CW) but also includes the *Mycoplasma* (devoided of a CW) since they also possess only one biological membrane. Monoderm bacteria are also called monodermata.	Shatalkin, 2004; Desvaux et al., 2009b; Sutcliffe, 2010; Gupta, 2011
Diderm-LPS bacteria	Bacterial species exhibiting two biological membranes and where the assymetrical OM contains lipopolysaccharide (LPS) on the external side.	Corresponds to the archetypal Gram-negative bacteria. Some diderm bacteria lack LPS in their OM and are called simple-diderm bacteria. Diderm bacteria are also called didermata.	Shatalkin, 2004; Desvaux et al., 2009b; Sutcliffe, 2010; Gupta, 2011
Diderm-mycolate bacteria	Bacterial species exhibiting two biological membranes and where an outer lipid layer called MOM is composed of mycolic acid molecules arranged in a highly ordered form.	Corresponds to the archetypal acid-fast bacteria, e.g., *Mycobacterium* and *Corynebacterium*.	Shatalkin, 2004; Desvaux et al., 2009b; Sutcliffe, 2010; Gupta, 2011
Secretome	Concept for an integrated and global view of the protein secretion by considering protein routing, transport mechanisms, post-translational modifications, and protein subcellular location.	The secretome considers both secreted proteins and proteins constituting the secretion machinery (and associated maturation pathways). This original definition of the secretome has been somehow usurped, misused, and misunderstood by some authors in the literature. The secretome can be investigated by different “omics” approaches (i.e., proteogenomics, transcriptomics, proteomics, and meta-omics counterparts) but is not a proteome *per se*. The secretome is not the exoproteome, which is the most commonly investigated but only one of the complement of the secretome. Other complements of the secretome can be (i) the protein secretion systems, (ii) the cell-surface proteins (including single and supramolecular protein structure), or (iii) the lipoproteome, etc… Since it is not secretion *per se*, the secretome do not cover protein release upon molecular events that are not active translocation process, e.g., cell lysis (autolysis, allolysis, bacteriophage lysis), or membrane budding (vesicles).	Tjalsma et al., 2000; Antelmann et al., 2001, 2006; Van Dijl et al., 2001; Economou, 2002; Sarvas et al., 2004; Buist et al., 2006; Desvaux et al., 2009b
Secretion	Active transport from the interior to the exterior of the cell.	Applies to protein entirely outside of the outer-most lipid bilayer, including exoproteins, surface proteins, and cell-surface appendages (e.g., pili and flagella, cellulosomes). In diderm bacteria, secretion is mediated by specific translocon for transport across the OM (or MOM in diderm-mycolate bacteria) and cannot be defined by the translocons located at IM (CM). *Sensu stricto*, it does not cover molecular events that are not active translocation process and thus not secretion *per se*, e.g., cell lysis (autolysis, allolysis, bacteriophage lysis) or membrane budding (vesicles).	Desvaux et al., 2004b, 2009b; Economou et al., 2006
Export	Active transport across the CM	In monoderm bacteria, export and secretion are synonymous but not in diderm bacteria. In diderm bacteria, the Sec and Tat translocon directs proteins to the CM (IM) or periplasm but cannot ensure their secretion *sensu stricto*.	Desvaux et al., 2004b, 2009b; Economou et al., 2006
Translocation	Active transport across a biological membrane.	Relative to the monoderm/diderm-LPS/diderm-mycolate bacteria trichotomy, it can occur at the CM (IM), OM, and/or MOM. Translocation event results in a change of subcellular location.	Economou et al., 2006; Desvaux et al., 2009b
Extracytoplasmic protein	Protein found outside the cytoplasm.	Corresponds to a protein either located elsewhere than the cytoplasm, e.g., intrinsic to a membrane (IMP, OMP, MOMP, lipoproteins), extrinsic to a membrane but on the side away from the cytoplasm, within the periplasm, associated to CW, at the cell surface or into the extracellular milieu. In other words, every protein except a cytoprotein. This subset of proteins corresponds to the extracytoproteome.	Dinh et al., 1994; Skorko-Glonek and Sobiecka-Szkatula, 2008
Exoprotein	Protein present in the extracellular milieu, i.e., an extracellular protein.	Corresponds to free soluble protein find in the extracellular milieu or within a host cell. Some exoproteins are not necessarily secreted *sensu stricto*, since it can occur by not active translocation process e.g., cell lysis (autolysis, allolysis, bacteriophage lysis), or membrane budding (vesicles).	Pugsley and Francetic, 1998; Desvaux et al., 2009b
Cytoprotein	Protein present in the cytoplasm, i.e., a cytoplasmic protein.	Corresponds to a cytosoluble protein, protein extrinsic to the CM located on the cytoplasmic side or a protein subunit of a CM protein complex but which subunit is entirely outside of the CM.	
Exoproteome	The subset of proteins present in the extracellular milieu (the exoproteins), i.e., the extracellular proteome.	It corresponds to one of the complements of the secretome.	Tjalsma, 2007; Desvaux et al., 2009b
Surface proteome	The subset of proteins present on the bacterial cell surface (the cell-surface proteins).	It corresponds to one of the complements of the secretome. The terminology “surface proteome” should be preferred to “surfaceome” (or “surfacome”) because it stresses it focuses on the protein content and not all the components on the bacterial cell surface (LPS, teichoic acids, exopolysaccharides, polyglutamate, etc…) as misleadingly suggested by the term “surfaceome.”	Cullen et al., 2005; Desvaux et al., 2006a, 2009b; Dreisbach et al., 2011; Voigt et al., 2012

a *IM, inner membrane; CM, cytoplasmic membrane; CW, cell wall; OM, outer membrane; MOM: mycolate outer membrane; IMP, integral membrane protein; OMP, outer membrane protein; MOMP, mycolate outer membrane protein*.

With these different concepts in hand, it becomes clear a comprehensive understanding of protein determinants involved in bacterial adhesion and/or biofilm formation necessitates a consideration of the cell envelope architecture, i.e., respective to the diderm-LPS, monoderm and diderm-mycolate bacteria trichotomy, as well as their respective protein secretion systems.

## The protein secretion systems in diderm-LPS, monoderm and diderm-mycolate bacteria

**Figure 2:**
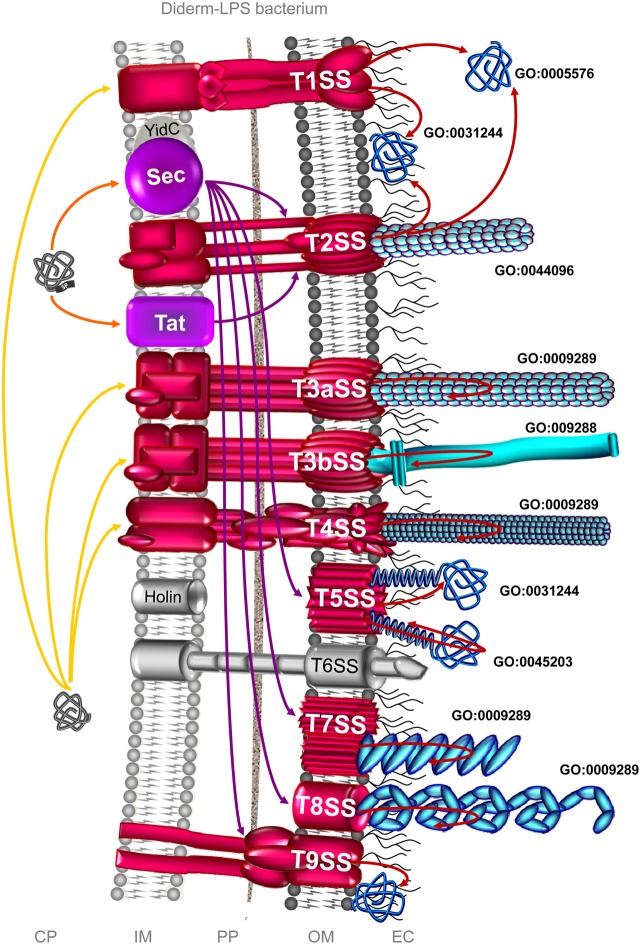
**The complement of the secretome involved in colonization process in diderm-LPS bacteria**. Among the 9 distinct secretion systems through which a secreted protein can be translocated across the OM in diderm-LPS bacteria, the T1SS, T2SS, T3SS, T4SS, T5SS, T7SS, T8SS, and T9SS can be involved in colonization process (depicted in red). Translocation machineries depicted in violet are protein export pathway participating to the protein transport of effectors involved in colonization process. The T1SS can secrete adhesins, which the release into the extracellular milieu (GO:0005576) and/or the association to the external side of the OM (GO:0031244) remain to be clarified. Besides the secretion of adhesion factors, the T2SS is involved in the formation of Type 4 pilus (GO:0044096), i.e., the T2SS subfamily c (T2cSS). The subfamily a of the T3SS (T3aSS) can be involved in the formation of pilus structure (GO:0009289), i.e., either the injectisome or the Hrp (hypersensitive response and pathogenicity) pilus, whereas the subfamily b of the T3SS (T3bSS) is involved in flagellum assembly (GO:009288). The T4SS is involved in the formation of pili (GO:0009289), either pilus T (T4aSS) or pilus F (T4bSS). The T5SS is involved in the secretion of adhesion either integral (GO:0045203) or extrinsic (GO:0031244) to the OM. The T7SS (CUP; chaperone-usher pathway) is involved in the formation of Type 1 pilus, and the T8SS (ENP; extracellular nucleation-precipitation pathway) in the formation of pilus of the type curli. The T9SS (Por secretion system) can secrete adhesins involved in gliding motility. In addition, some surface proteins could use systems as yet uncovered, the so-called non-classical (NC) secretion. Only branches corresponding to the complement of the secretome involved in bacterial colonization are colored. Extracytoplasmic proteins, i.e., single proteins and supramolecular protein structures, potentially involved in surface colonization are depicted in blue. Orange and yellow arrows indicate the routes for proteins targeted to the CM possessing or lacking an N-terminal SP, respectively. Violet arrows indicate the routes for exported proteins and red arrows for secreted proteins. CP, cytoplasm; IM, inner membrane; PP, periplasm; OM, outer membrane; EC, extracellular milieu; SP, signal peptide.

In diderm-LPS bacteria, nine protein secretion systems have been unravelled so far. For better or worse, these systems have been numerically classified from the Type I (T1SS) to Type IX secretion systems (T9SS) (Desvaux et al., 2009b; McBride and Zhu, 2013) (Figure 2). The T1SS refers to a three-component complex, i.e., a pore-forming OM protein (OMP) of the TolC family, a membrane fusion protein (MFP), and an IM ATP-binding cassette (ABC) exporter (Delepelaire, 2004; Holland et al., 2005; Lee et al., 2012); a common misunderstanding is to make the T1SS synonymous to an ABC transporter (Desvaux et al., 2009b). The T2SS, also called the secreton-depend pathway (SDP) is a protein complex composed of around a dozen of proteins bridging the IM and OM to allow secretion of proteins translocated in the first place by the Sec or Tat export system (Sandkvist, 2001; Voulhoux et al., 2001; Cianciotto, 2005; Douzi et al., 2012; McLaughlin et al., 2012). It can be stressed again that referring to the general secretory pathway (GSP) or to the main terminal branch (MTB) for this system is nowadays obsolete and misleading (Desvaux et al., 2004b); as referred to in Pfam (Bateman et al., 2004), the naming of the different T2SS subunits as T2SE for instance is much more preferable than GspE (Peabody et al., 2003; Desvaux et al., 2004b). The T3SS is a highly complex molecular machine composed of at least 20 proteins and also one of the most extensively investigated protein secretion system (Ghosh, 2004; Cornelis, 2006, 2010; Minamino et al., 2008; Büttner, 2012). The T4SS is composed of around a dozen of proteins subunits forming a protein-conducting channel spanning the entire bacterial cell envelope (Christie et al., 2005; Waksman and Fronzes, 2010; Zechner et al., 2012); there is still much misunderstanding and confusion in part of the scientific literature about the T4SS and T2SS, as well as Type 4 pili (T4P), but it must be stressed again they can be clearly phylogenetically differentiated (Nunn, 1999; Planet et al., 2001; Mattick, 2002; Peabody et al., 2003; Hazes and Frost, 2008). T5SS refers to proteins depending on the Sec machinery for IM transit and then transported across the OM *via* a translocation unit formed by a β-barrel to complete secretion (Henderson et al., 2004; Leo et al., 2012); the BAM (β-barrel assembly machinery) complex as well as several periplasmic chaperones (namely SurA, Skp, DegP, and FkpA) are taking part to the secretion process across the OM (Desvaux et al., 2004a; Knowles et al., 2009; Ruiz-Perez et al., 2010; Rossiter et al., 2011b; Leyton et al., 2012). The T6SS is a composite system of at least 13 protein subunits from various hypothetical phylogenetic origins, with essentially two subassemblies, i.e., one dynamic structure related the contractile bacteriophage tail-like structure and one cell-envelope-spanning membrane-associated complex (Cascales and Cambillau, 2012; Silverman et al., 2012). The T7SS corresponds to the chaperone-usher pathway (CUP) used for pilus assembly (Desvaux et al., 2009b; Waksman and Hultgren, 2009; Busch and Waksman, 2012); as further explained below, this system for diderm-LPS bacteria must not be mistaken with the diderm-mycolate bacterial “Type VII secretion system,” which is in fact the ESX (ESAT-6 system). The T8SS corresponds to the extracellular nucleation-precipitation pathway (ENP) (Barnhart and Chapman, 2006; Desvaux et al., 2009b; Blanco et al., 2012; Dueholm et al., 2012). The T9SS corresponds to the Por (porphyrin accumulation on the cell surface) secretion system (Sato et al., 2010, 2013; Shoji et al., 2011; McBride and Zhu, 2013). In diderm-LPS bacteria, the complement of the secretome potentially involved in bacterial colonization process gathers some secreted proteins and their associated secretion systems, which can be either the T1SS, T2SS, T3SS, T4SS, T5SS, T7SS, T8SS, or T9SS (Figure 2). In general, the secreted proteins involved in bacterial colonization are either cell-surface exposed single proteins or subunits of cell-surface supramolecular complexes, such as pili or flagella. In the rest of the manuscript, pili will be used as a generic term synonymous with fimbriae or curli (which are just some particular types of pili).

**Figure 3:**
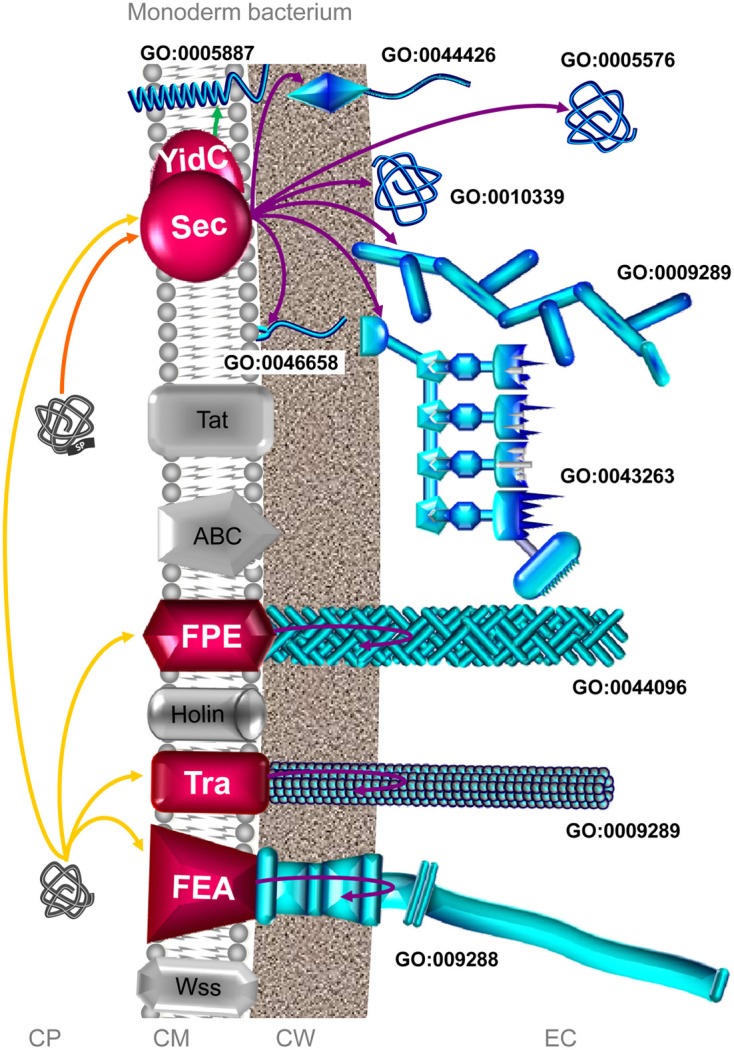
**The complement of the secretome involved in colonization process in monoderm bacteria**. Among the 8 distinct secretion systems through which a secreted protein can be translocated across the CM, the Sec, FPE, Tra, and FEA pathways can potentially be involved in colonization process in monoderm bacteria (depicted in red). The Sec pathway covers (i) integration of membrane protein (GO:0005887) *via* YidC, (ii) the anchoring to CM (GO:0046658) *via* the lipoprotein maturation pathway, (iii) the anchoring to the CW (GO:0044426) in a covalent or non-covalent manner, (iv) the association on the external side of the CW (GO:0010339), (v) the formation of cell surface supramolecular structure, namely pilus (GO:0009289) and cellulosome (GO:0043263), and (vi) protein secretion in the extracellular milieu (GO:0005576). It is worth noting that some proteins with no N-terminal SP can be translocated *via* Sec in a SecA2-dependent manner in monodermata (Rigel and Braunstein, 2008; Renier et al., 2013). The FPE is involved the formation of Type 4 pilus (GO:0044096). The Tra system (misleadingly called “Type IV-like secretion system” in monoderm bacteria) is involved in the formation of conjugative pili (GO:0009289). The FEA is involved in the secretion and assembly of the flagellum protein subunits (GO:009288). In addition, some surface proteins could use systems as yet uncovered, the so-called non-classical (NC) secretion. Extracytoplasmic proteins, i.e., single proteins and supramolecular protein structures, potentially involved in surface colonization are depicted in blue. Only branches corresponding to the complement of the secretome involved in bacterial colonization are colored. Orange and yellow arrows indicate the routes for proteins targeted to the CM possessing or lacking an N-terminal SP. Violet arrows indicate the routes for exported/secreted proteins (export and secretion are synonymous in monoderm bacteria). Green arrow indicates proteins integrated into the CM. CP: cytoplasm; CM: cytoplasmic membrane; CW: cell wall; EC, extracellular milieu; SP: signal peptide.

There are no counterparts to the molecular machineries required for transport across the OM of diderm bacteria in monoderm bacteria as this membrane is not present in the latter organisms. Consequently, the use of the numerical classification for systems dedicated to protein secretion in diderm-LPS bacteria (i.e., protein transport from inside to outside the cell across the IM and OM) does not make any sense and cannot be applied to monoderm bacteria. However, this does not prohibit phylogenetic relationships between the protein translocation systems in monoderm and diderm-LPS bacteria. Indeed, they both possess a cytoplasmic membrane (also called IM in didermata) with some common protein transport systems allowing secretion in monoderm bacteria and export in diderm-LPS bacteria, respectively. The protein secretion system present in monoderm bacteria are (i) the Sec (secretion), (ii) the Tat (twin-arginine translocation), (iii) ABC protein exporter, (iv) the FPE (fimbrilin-protein exporter), (v) the holin (hole forming), (vi) the Tra (transfer), misleadingly called the “Type IV-like secretion system” in monoderm bacteria), (vii) the FEA (flagella export apparatus), and (viii) the Wss (WXG100 secretion system) (Desvaux et al., 2009b; Desvaux, 2012) (Figure 3). From the current knowledge in the field, the complement of the secretome potentially involved in the colonization process in monoderm bacteria gathers the Sec, FPE and FEA as well as some of their respective substrates (Figure 3). These systems secrete either cell-surface exposed single proteins or subunits of cell-surface supramolecular complexes, such as pili, cellulosome, or flagella (Renier et al., 2012).

**Figure 4:**
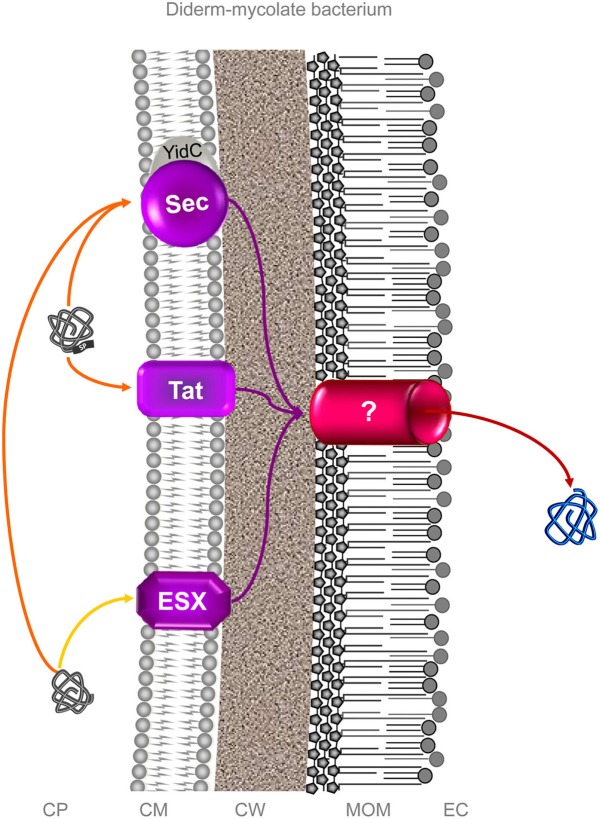
**Protein secretion in diderm-mycolate bacteria**. In diderm-mycolate bacteria, three protein export systems are currently recognized, the Sec, Tat and ESX (ESAT-6 system) (Digiuseppe Champion and Cox, 2007; Feltcher et al., 2010; Ligon et al., 2012). So far, none have been clearly reported and characterized as involved in surface colonization process. As in monodermata, some proteins with no N-terminal SP can be translocated *via* Sec in a SecA2-dependent manner (Feltcher and Braunstein, 2012). While it is clear some exported proteins (i.e., first translocated across the CM by these export systems) are further secreted into the extracellular milieu (i.e., translocated across the MOM), no mycomembrane machinery allowing the translocation across the MOM have been identified to date (Niederweis et al., 2010; Houben et al., 2012; Ligon et al., 2012; Freudl, 2013; Van Der Woude et al., 2013). In other no words (Table 1), no protein secretion system has been *sensu stricto* identified as yet in diderm-mycolate bacteria. It is still an enigma whether one MOM translocon or specific translocons for each of the three protein export systems are present or if the protein secretion is completed in a one-step or two-steps process (Desvaux et al., 2009a). This (or those) MOM translocon would truly correspond to a protein secretion system. For these different reasons and others, numbering the ESX (as “Type VII secretion system”) in diderm-mycolate bacteria is clearly premature and misleading (Desvaux et al., 2009a,b). Orange and yellow arrows indicate the routes of proteins targeted to the CM possessing or lacking an N-terminal SP. Violet arrows indicate the routes for exported proteins and red arrows for secreted proteins. CP, cytoplasm; CM, cytoplasmic membrane; CW, cell wall; MOM, mycolate outer membrane (or mycomembrane); EC, extracellular milieu; SP, signal peptide.

In diderm-mycolate (archetypal acid-fast) bacteria, the term “Type VII secretion system” has also been coined to describe a Wss-like machinery (Economou et al., 2006; Abdallah et al., 2007; Schneewind and Missiakas, 2012). It must be stressed, this “Type VII secretion system” is restricted to diderm-mycolate bacteria only since this system is absolutely not found in any archetypal diderm-LPS bacteria and, consequently, does not fit with standard numerical classification of protein secretion systems specifically designated for diderm-LPS (archetypal Gram-negative) bacteria (Salmond and Reeves, 1993; Sutcliffe, 2010). This “Type VII secretion system” further stands alone in diderm-mycolate bacteria since there are neither T1SS, T2SS, T3SS, T4SS, T5SS, nor T6SS but only Sec and Tat systems (which are not included in the standard numerical classification) (Digiuseppe Champion and Cox, 2007) (Figure 4). The molecular machinery described for this diderm-mycolate bacterial “Type VII secretion system” is an export pathway (protein transport across the cytoplasmic membrane) just as the Sec and Tat systems are (Economou et al., 2006; Desvaux et al., 2009a; Houben et al., 2012). In fact, no translocon in the mycolate outer membrane (MOM), which would truly enable protein secretion and thus form a complete secretion pathway, has been uncovered as yet in diderm-mycolate bacteria [the secretion of proteins exported in the first instance by the Wss, Sec, and Tat could then be completed by the very same MOM translocon, or different MOM translocons specific to each of these export systems (Desvaux et al., 2009a)] (Niederweis, 2003; Converse and Cox, 2005; Ize and Palmer, 2006; Digiuseppe Champion and Cox, 2007; Song et al., 2008; Desvaux et al., 2009a; Niederweis et al., 2010; Stoop et al., 2012; Freudl, 2013; Van Der Woude et al., 2013). This “Type VII secretion system” nomenclature in diderm-mycolate bacteria is clearly not compatible with the numerical classification basically designed to describe OM translocation systems in diderm-LPS bacteria (Salmond and Reeves, 1993; Economou et al., 2006; Desvaux et al., 2009a). All-in-all, using the “Type VII secretion system” denomination for the phylogenetically related secretion systems in monoderm bacteria (i.e., the Wss) is very much confusing since it does not align with the other secretion systems present such as Sec and Tat (which do not withstand the numerical classification terminology) (Desvaux et al., 2004b, 2009a). When designating this “Type VII secretion system” it is then highly advisable to clearly specify it relates to diderm-mycolate bacteria only to prevent any confusion with the unrelated T7SS in diderm-LPS bacteria. Ultimately, its use should be refrained in favor of the “ESX (ESAT-6 system)” designation in diderm-mycolate bacteria (archetypal acid-fast bacteria) and/or the generic “WXG100 secretion system (Wss)” designation especially relevant to monodermata (archetypal Gram-positive bacteria). At the moment only protein export systems have been reported in diderm-mycolate bacteria (Digiuseppe Champion and Cox, 2007; Feltcher et al., 2010; Ligon et al., 2012) but *sensu stricto* no protein secretion system has been identified (Figure 4). As for the T1SS to T9SS in diderm-LPS bacteria, only the identification of translocon components at the MOM would truly permit to define a protein secretion system (Desvaux et al., 2009a), which at worse could be the unique terminal branch for all the three export systems (Sec, Tat, and ESX) in diderm-mycolate bacteria (Figure 4).

For effectors involved in bacterial colonization, this review will especially focus on protein secretion systems in diderm-LPS (archetypal Gram-negative) and monoderm (archetypal Gram-positive) bacteria since none has been characterized as yet in diderm-mycolate (archetypal acid-fast) bacteria.

## Secreted proteins involved in surface colonization in diderm-LPS bacteria

Out of the 9 distinct protein secretion systems through which a secreted protein can be translocated across the OM, only the T6SS has never been reported so far to be involved in bacterial adhesion and/or biofilm formation in diderm-LPS bacteria (Figure 2). The T2SS, T3SS, T4SS, and T5SS are further divided into different subtypes.

### Adhesins secreted via the T1SS

The T1SS has been demonstrated to allow the secretion of large bacterial adhesins belonging to the Bap family (Delepelaire, 2004; Lasa and Penadés, 2006; Latasa et al., 2006). In *Salmonella enterica*, SiiE (*Salmonella* intestinal infection E) and BapA (Biofilm-associated protein A) have been characterized. SiiE is a very large adhesin of 600 kDa of *Salmonella enteric* required for adhesion to epithelial cells. SiiE would essentially exist as an exoprotein that may only be loosely associated the OM but upon host cell contact it is retained on the bacterial cell surface (Wong et al., 1998; Morgan et al., 2004; Gerlach et al., 2007). The ability of SiiE to bind Ca^2+^ ions would confer a rigid rod-like habitus that is required for reach out beyond the LPS and initiates bacterial adhesion to polarized host cells (Wagner et al., 2011; Griessl et al., 2013). BapA is a 386 kDa protein allowing homotypic adhesion during *Salmonella* biofilm formation and is involved in pathogenesis, especially internalization and invasion of intestinal epithelium (Latasa et al., 2005; Jonas et al., 2007; Biswas et al., 2011; Suez et al., 2013). BapA is secreted extracellularly where it remains in loose association with the bacterial cell surface. In *Pseudomonas fluorescens*, LapA (large adhesion protein A) is the largest T1SS-dependent adhesin uncovered so far, with a molecular weight estimated at 888 kDa (Hinsa et al., 2003; Hinsa and O'toole, 2006). LapA enables irreversible adhesion and biofilm formation on abiotic surfaces but also adhesion to corn seeds in *Pseudomonas putida* (Mus20 or Mus24; mutant unattached to seeds) (Espinosa-Urgel et al., 2000; Huber et al., 2002; Hinsa et al., 2003; Ivanov et al., 2012; Zhang et al., 2013). LapA is found both in the extracellular milieu and in a loose association with the bacterial cell surface but not in the OM (Hinsa et al., 2003). In *Burkholderia cepacia*, the loss of Bap resulted in decreased surface hydrophobicity and in colony conversion to a rough morphotype (Huber et al., 2002). Additional adhesins of the Bap family, presumably secreted *via* a T1SS as supported by the genomic context, have been characterized in diderm-LPS bacteria, namely VPA1445 (*Vibrio parahaemolyticus* locus 1445) (Enos-Berlage et al., 2005) and YeeJ (systematic nomenclature) in *Escherichia coli* (Roux et al., 2005). In all cases, the mediation of adhesion *via* an extracellular protein is difficult to conceptualize and still demands experimental investigations for validation (Gerlach and Hensel, 2007).

### Type 4 pilus (T4P) and colonization factor *via* the T2SS

Based on phylogenetic analysis, T2SSs were further subdivided into the subfamilies T2aSS and T2bSS, corresponding, respectively, to Xcp (*Xanthomonas campestris* general secretion pathway) and Hxc (homolog to Xcp) systems (Filloux, 2004; Michel and Voulhoux, 2009; Durand et al., 2011). At the same period, it was proposed to include the Type 4 piliation (T4P) system (Desvaux et al., 2009b) within the T2SS since the T4P is homologous to the Xcp and falls into discrete phylogenetic cluster (Planet et al., 2001; Peabody et al., 2003; Tomich et al., 2007; Ayers et al., 2010). To make the nomenclature coherent, we propose to classify those systems as T2aSS for the classical SDP extensively investigated in *Pseudomonas aeruginosa*, i.e., Xcp type system (Voulhoux et al., 2001), T2bSS for the Hxc type system (Durand et al., 2011), and T2cSS for the T4P system (Mattick, 2002). The Type 4 pili promote initial bacterial attachment and are of importance for the early stages of biofilm development, i.e., microcolony formation (Hahn, 1997; Giltner et al., 2006; Burrows, 2012). The Type 4 pili are subdivided into two main classes assembled by the T2cSS (Strom and Lory, 1993; Kachlany et al., 2001; Pelicic, 2008), i.e., (i) the T4a pilins, and (ii) the T4b pilins (Strom and Lory, 1993; Skerker and Shapiro, 2000; Craig et al., 2004; Craig and Li, 2008; Giltner et al., 2012). Besides adherence, the T4aP are responsible for a number of related processes associated with bacterial motility, including twitching, swarming, crawling, walking, and slingshoting, which participate to biofilm development (Desvaux et al., 2005a; Burrows, 2012; Conrad, 2012).

The colonization factor GbpA (N-acetylglucosamine-binding protein A) from *Vibrio cholerae* is secreted in a T2SS-dependent manner in the extracellular milieu (Kirn et al., 2005). This protein is required for efficient environmental colonization by organisms such as zooplankton, and also intestinal colonization, especially of human epithelial cells, by binding to a sugar present on both surfaces (Stauder et al., 2012). GbpA has a modular architecture with chitin-binding and mucin-binding domains enabling attachment to different host surfaces and other domains binding to the bacterial cell surface (Wong et al., 2012). Despite its secretion into the extracellular milieu, GbpA thus has a bridging function between *V. cholerae* and its host allowing efficient colonization of chitinous exoskeletons of arthropods or the intestinal epithelium.

### T3SS: injectisome, Hrp (hypersensitive response and pathogenicity) pilus and flagellum

The T3SSs can be subdivided into (i) the non-flagellar T3SS, i.e., the T3aSS, involved in the assembly of the injectisome or Hrp (hypersensitive response and pathogenicity) pilus, and (ii) the flagellar T3SS, i.e., T3bSS, responsible for assembly of the flagellum (Tampakaki et al., 2004; Journet et al., 2005; Pallen et al., 2005; Desvaux et al., 2006b).

While acting primarily as a molecular syringe for injecting protein effectors directly into the cytosol of a host cell (Cornelis, 2006, 2010), the injectisome (T3aSS) mediates intimate bacterial adhesion and colonization of cells such as gut epithelial cells (Garmendia et al., 2005). In enterohemorrhagic *Escherichia coli* (EHEC), it was further demonstrated to play a role in the adhesion to lettuce leaves with a marked tropism for the stomata, and thus to contribute to the transmission of this food-borne pathogen to humans (Shaw et al., 2008; Berger et al., 2010). While the injectisome is found in animal-pathogenic bacteria, the Hrp pilus (T3aSS) is found in plant-pathogenic bacteria (Tang et al., 2006; Tampakaki et al., 2010). By injecting T3SS effectors into the host cell, the Hrp pilus allows the phytopathogens to subdue the vegetable-cell response (Zhou and Chai, 2008) but also participate to plant colonization through initial bacterial adhesion (Darsonval et al., 2008; Correa et al., 2012).

The flagella (T3bSS) are involved in cell motility through swimming and/or swarming, where it can contribute to bacterial colonization by bringing the cell in contact with biotic or abiotic surfaces by chemiotaxis for instance, or by translocating the bacterial cells over a surface, respectively (Pratt and Kolter, 1998; Harshey, 2003; Verstraeten et al., 2008). Besides, the flagella mediate bacterial adhesion directly to biotic and abiotic surfaces, e.g., to plasticware, human mucus and mucin, epithelial cells, enterocytes, vegetables (Grant et al., 1993; Ramphal et al., 1996; Lillehoj et al., 2002; Kirov et al., 2004; Berger et al., 2009b; Mahajan et al., 2009; Shaw et al., 2011; Tran et al., 2011; Bucior et al., 2012; Troge et al., 2012). Interestingly in *Salmonella enterica*, adhesion to salad leaves mediated by the flagella was shown to be strain-specific (Berger et al., 2009a). *Salmonella enterica* can further internalize into vegetable leaves through flagella-chemotaxis via open stomata (Kroupitski et al., 2009a). Although the internalization is variable in leafy vegetables and fresh herbs (Golberg et al., 2011; Kroupitski et al., 2011), the flagella is clearly an important determinant for colonization of some vegetables and can thus contribute to the transmission of this food-borne pathogen to humans even after washing (Berger et al., 2009a; Kroupitski et al., 2009a,b).

### Pili assembled by the T4SS

The T4SSs are broadly subdivided into (i) T4aSS corresponding to the prototypical VirB/D4 complex extensively investigated in *Agrobacterium tumefaciens*, and (ii) T4bSS corresponding to the prototypical F-conjugal transfer system of the self-transmissible IncI plasmid composed of the widely conserved Tra (transfer) proteins also present in monoderm bacteria (Christie and Vogel, 2000; Sexton and Vogel, 2002; Lawley et al., 2003; Harris and Silverman, 2004; Christie et al., 2005; Hazes and Frost, 2008; Voth et al., 2012). The T4SS allows transport of proteins into prokaryotic or eukaryotic cells. This system is also involved and ancestrally related to bacterial conjugation with the transport of DNA as nucleo-protein complex (Lawley et al., 2003). Rather than focusing on protein secretion system only, the nomenclature is quite confusing at the moment since it mixes up homologic (phylogenetic) and analogic (functional) based-classifications on aspects related to DNA transport as well as the systems present in monoderm and diderm-LPS bacteria (Alvarez-Martinez and Christie, 2009; Wallden et al., 2010); in addition, some phylogenetic relatedness between the T2SS assembling Type 4 pili (T4P) and T4SS assembling conjugative pili of Type 2 (Ottow, 1975) accentuates the complexity of the situation (Hazes and Frost, 2008). As it has been done previously for other protein secretion systems (Henderson et al., 2000; Desvaux et al., 2004b, 2006b, 2009b), this stresses the need to clarify the classification within the T4SS respective to the current terminology and ontology issues in the field of bacterial protein secretion, which should be rather based on homology and phylogeny rather than analogy (Desvaux et al., 2006b, 2009b). Regarding bacterial colonization, the F episome (natural conjugative plasmid) was demonstrated to induce the formation of a thick biofilm in *E. coli* and that conjugative pilus synthesis from the *tra* operon of the F plasmid was required for biofilm formation (Ghigo, 2001). In fact, the bacterial cells harboring the F episome had increased adhesion ability thanks to the expression of conjugative pili. It appeared these plasmids can be further transferred by conjugation from these donor cells to recipient cells. Then, the increasing proportion of transconjugant cells within the biofilm further improved the colonization ability of the bacterial population. While the positive influence of conjugative plasmid on biofilm formation was reported in other species (Bahl et al., 2007; Burmolle et al., 2008), it can also have adverse effects in complex multi-species biofilms and would depend on the composition of the bacterial community (Roder et al., 2013).

Phylogenetic analyses revealed the tight-adherence (Tad) system originally uncovered in *Aggregatibacter* (formerly *Actinobacillus*) *actinomycetemcomitans* was actually related to the T4SS and would constitute a major subfamily (Tomich et al., 2007). The Tad system is involved in the piliation of the Flp (fimbrial low-molecular-weight protein). Those pili are composed of Type 4 pilin of the subfamily b (Giltner et al., 2012). Flp pili are highly adhesive and essential for biofilm formation (Kachlany et al., 2001). Rather than binding to specific receptor(s), the bacterial–bacterial and bacterial–host interactions lead to strong attachment. Bacterial aggregation is mediated by the association of pilus fibers, which leads to pilus bundling. This system is widespread among diderm-LPS bacterial species, including the genera *Haemophilus, Pasteurella, Pseudomonas, Yersinia*, or *Caulobacter* (Kachlany et al., 2000, 2001; Bernard et al., 2009).

### Cell-surface adhesins of the T5SS

T5SSs are subdivided into five subtypes (Henderson et al., 2000, 2004; Desvaux et al., 2003, 2004a; Henderson and Desvaux, 2004; Salacha et al., 2010; Leo et al., 2012), (i) the T5aSS corresponding to the classical autotransporter or autotransporter of Type 1 (AT-1), (ii) the T5bSS corresponding to the two-partner secretion (TPS), (iii) the T5cSS corresponding to the trimeric autotransporter or autotransporter of Type 2 (AT-2), (iv) the T5dSS corresponding to hybrid autotransporter between AT-1 and TPS, or autotransporter of Type 3 (AT-3), and (v) the T5eSS corresponding to invasin/intimin family of inverted autotransporter or autotransporter of Type 4 (AT-4). No function in bacterial colonization has been reported as yet for the hybrid autotransporters (T5dSS) (Salacha et al., 2010).

#### Adhesins of the classical autotransporter pathway (T5aSS)

Among the classical autotransporters (T5aSS), the SAATs (Self-associating autotransporters) actively participate in biofilm development and encompasse the Ag43 (antigen 43), AIDA (adhesin involved in diffuse adherence,) and TibA (enterotoxigenic invasion locus b protein A) (Klemm et al., 2006; Van Der Woude and Henderson, 2008). The SAATs promote bacterial auto-aggregation and interaction with other SAAT partners, for instance between AIDA and Ag43, leading to the formation of mixed bacterial cell aggregates (Sherlock et al., 2004). Self-recognition is sensitive to environmental conditions, such as pH or the presence of bile salt (Klemm et al., 2004; Sherlock et al., 2004, 2005; Girard et al., 2010) and intercellular aggregation can be abolished by pili or exopolymers, which compromise SAATs interaction (Klemm et al., 2006). SAATs further mediate and enhance biofilm formation (Klemm et al., 2006). Ag43, however, would not contribute significantly to intestinal colonization (Parham et al., 2005; De Luna et al., 2008). Besides SAATs, several other AT-1s act as adhesins (Henderson et al., 2004; Girard and Mourez, 2006). YfaL, YpjA, and YcgV (*E. coli* systematic nomenclature) are involved in initial bacterial adhesion and biofilm formation (Roux et al., 2005). BrkA (*Bordetella* resistance to killing protein A) (Ewanowich et al., 1989; Fernandez and Weiss, 1994) and Pertactin (Leininger et al., 1991) promote adhesion to different mammalian cells. Aae (*A. actinomycetemcomitans* epithelial cell binding) (Rose et al., 2003; Fine et al., 2005), App (adhesion and penetration protein) (Serruto et al., 2003), CapA (*Campylobacter* adhesion protein A) (Ashgar et al., 2007), EspP (extracellular serine protease P) (Dziva et al., 2007; Puttamreddy et al., 2010), McaP (*Moraxella catarrhalis* adherence protein) (Timpe et al., 2003; Lipski et al., 2007), PmpD (Polymorphic membrane protein D) (Wehrl et al., 2004), rOmpB (Rickettsia outer membrane protein B) (Uchiyama et al., 2006), and Sab (STEC autotransporter mediating biofilm formation) (Herold et al., 2009), enable adherence to epithelial cells; for some of them it was further demonstrated they participated to intestinal colonization and/or biofilm formation, namely CapA, EspP and Sab. Hap (*Haemophilus* autotransporter protein) further mediates microcolony formation as well as binding to fibronectin, laminin, and collagen (Fink et al., 2003), whereas MisL (membrane insertion and secretion protein L) and ShdA (shedding protein A) bind to collagen and fibronectin (Kingsley et al., 2000, 2002, 2004; Dorsey et al., 2005). EhaA (EHEC autotransporter A) (Wells et al., 2008), EhaB (Wells et al., 2009), and EhaJ (Easton et al., 2011) are MSCRAMM (microbial surface components recognizing adhesive matrix molecules) proteins binding differentially to various extracellular matrix (ECM) proteins and contributing to biofilm formation (Chagnot et al., 2012, 2013). Tsh (temperature-sensitive haemagglutinin) also binds to ECM proteins and further adheres to red blood cells and hemoglobin (Kostakioti and Stathopoulos, 2004). AlpA (adherence-associated lipoprotein A) (Odenbreit et al., 1999), BabA (blood group antigen–binding adhesin) (Ilver et al., 1998), and SabA (sialic acid–binding adhesin) (Mahdavi et al., 2002) are involved in adhesion to human gastric epithelia by the fucosylated Lewis b histo-blood group antigen for BabA and by sialyl-dimeric-Lewis × glycosphingolipid for SabA.

Of major importance in the colonization process, the expression of many autotransporters (such as Ag43) is subjected to phase variation (Henderson et al., 1999; Van Der Woude and Henderson, 2008; Rossiter et al., 2011a). In addition, proteolytic processing of the passenger domain of the autotransporter on the bacterial cell surface can have a regulatory function on bacterial adhesion (Leyton et al., 2012). In fact, the passenger domain of AT-1s can either remain attached or cleaved off the translocation unit upon OM translocation. This proteolytic cleavage can arise following different scenarios (Leyton et al., 2012) but basically the cleavage can either be intramolecular and autocatalytic or intermolecular resulting in the release of passenger domain into the extracellular milieu, for example the SPATEs (serine protease autotransporters of *Enterobacteriaceae*). Some processed AT-1s, though, remain strongly associated with their cognate translocation unit, e.g., the adhesins Hap, pertactin, AIDA, or Ag43. It also appeared, the protein function is not modified when the cleavage is abolished (Charbonneau et al., 2009), which questions the purpose or benefit of this processing. For Hap, however, it was demonstrated the cleavage influenced the adherence to epithelial cells as well as adhesion to ECM proteins (Fink et al., 2002, 2003). Interestingly, NalP (*Neisseria* autotransporter lipoprotein), which is subjected to phase-variable expression by slipped-strand mispairing (Saunders et al., 2000) and modifies the cleavage patterns of other surface-associated proteins (including some other autotransporters and surface-exposed lipoproteins) (Van Ulsen et al., 2003, 2006; Roussel-Jazede et al., 2010; Serruto et al., 2010), affects in turn adhesion and biofilm formation (Arenas et al., 2013). Similar proteins are found in other bacterial pathogens.

#### Adhesins of the TPS pathway (T5bSS)

In the TPS (T5bSS), the passenger domain or exoprotein (TpsA) is translated separately from its cognate translocation unit (TpsB) (Jacob-Dubuisson et al., 2004). The exoprotein FHA (filamentous haemagglutinin) is a multifaceted adhesin and major attachment factor of *Bordetella* spp required for colonization of the lower respiratory tract (Locht et al., 1993). While part of the exoprotein is found to be surface-associated, another part is released into the extracellular milieu. Contrary to our previous understanding, it appeared that the C-terminus of FHA is oriented away from the bacterial cell surface and is required for adherence to epithelial cells (Mazar and Cotter, 2006). The exoproteins HMW1 (high-molecular-weight protein 1) and HMW2 of *Haemophilus influenza* are crucial colonization factors involved in bacterial adherence to a variety of respiratory epithelial cell types (St Geme et al., 1993; St Geme and Yeo, 2009). HrpA (hemagglutinin/hemolysin-related protein A) from *Neisseria meningitidis* and MhaB1 (*M. catarrhalis* FhaB-like protein 1) from *Moraxella catarrhalis* mediate binding to human epithelial cells (Schmitt et al., 2007). In the phytopathogen *Xanthomonas axonopodis*, XacFhaB (*X. axonopodis* pv. *citri* filamentous haemagglutinin-like protein B) is required for leaf tissue colonization, especially adhesion and biofilm formation (Gottig et al., 2009). In *Pseudomonas putida*, HlpA (haemolysin-like protein A) would play a direct role in the bacterial cell-root surface interaction (Molina et al., 2006). HecA (hemolysin-like *E. chrysanthemi* protein A) from *Erwinia chrysanthemi* contributes to the attachment and cell aggregation on leaves (Rojas et al., 2002). Interestingly, the exoprotein EtpA (ETEC two-partner secretion protein A) mediates bacterial adhesion by bridging the flagella with the host cells (Roy et al., 2009).

#### Adhesins of the trimeric autotransporter pathway (T5cSS)

A cardinal feature of the T5SS is a functional OM translocation unit, e.g., located at the C-terminus in monomeric classical autotransporter (T5aSS) or the TspB for the T5bSS. In the T5cSS, the C-terminal translocation unit is formed upon trimerization and serves as an OM anchor for cell-surface exposure of the passenger domains (Cotter et al., 2005; Lyskowski et al., 2011). YadA (*Yersinia* adhesin A) is the prototypical member of this T5SS subfamily (Hoiczyk et al., 2000; El Tahir and Skurnik, 2001; Eitel and Dersch, 2002; Nummelin et al., 2004; Leo et al., 2010). The high-molecular weight trimer formed by the passenger domains protrudes from the OM and consists of three domains, the stalk, necks and head (Linke et al., 2006; Hartmann et al., 2012). Differences in the size of the protruding AT-2s result from the length of their stalks (Linke et al., 2006). The head is involved in binding to some ECM proteins, including collagen, and makes YadA a major adhesion factor. All trimeric autotransporters (T5cSS) characterized to date are involved in bacterial adhesion (Linke et al., 2006). Besides YadA, several other AT-2s have been characterized to date in this expanding protein family (Cotter et al., 2005), e.g., Hia (*Haemophilus influenzae* adhesin) (Hoiczyk et al., 2000; St Geme and Cutter, 2000), UspA (ubiquitous surface protein A) of *Moraxella catharralis* (Conners et al., 2008), Hag (hemagglutinin) (Pearson et al., 2002; Bullard et al., 2007), NadA (*Neisseria* adhesin A) (Comanducci et al., 2002), BadA (*Bartonella henselae* adhesin) (Riess et al., 2004), NcaA (necessary for collagen adhesion A) (Fulcher et al., 2006), NhhA (*Neisseriahia* Hsf homologue protein A) (Scarselli et al., 2006), DsrA (*ducreyi* serum resistance A) (Leduc et al., 2008), EhaG (EHEC adhesin G) (Valle et al., 2008), SadA (*Salmonella* adhesin A) (Raghunathan et al., 2011), or UpaG (UPEC adhesin G) (Totsika et al., 2012). Each of these trimeric autotransporters are quite multifunctional and in terms of bacterial colonization they can be involved in autoagglutination, hemagglutination, ECM-binding, and/or adhesion to epithelial cells (El Tahir and Skurnik, 2001; Linke et al., 2006).

#### Intimins/invasins of the inverted autotransporter pathway (T5eSS)

Intimins/invasins (T5eSS) belong to a large and novel subfamily of the T5SS (Tsai et al., 2010; Leo et al., 2012; Oberhettinger et al., 2012). Compared to the classical autotransporter, the secretion mechanism is inverted in the sense, the translocation unit is located at the N-terminal instead of the C-terminal end of the monomeric autotransporter. The intimin/invasin family regroups adhesins that mediate bacterial adhesion and/or invasion of their host cells. In *Yersinia* spp., invasin binds to mammalian cell receptors of the integrin family (Isberg et al., 2000; Palumbo and Wang, 2006). In enteropathogenic *E. coli*, intimin mediates intimate adherence between the bacteria and the host cells by interacting with Tir (translocated intimin receptor) (Frankel and Phillips, 2008). Tir is a T3aSS-secreted protein, which integrates into the plasma membrane of the host cell (Devinney et al., 1999); upon binding to intestinal epithelial cells, the intimin-Tir interaction leads to the formation of actin pedestals beneath bound bacteria (Campellone and Leong, 2003; Brady et al., 2011). Interestingly, intimin can further promote intestinal colonization in a Tir-independent way (Mallick et al., 2012).

### Pili assembled by the T7SS

In diderm-LPS bacteria, the T7SS corresponds to the chaperone-usher pathway (CUP) (Desvaux et al., 2009b); as explained above it has nothing to do with the “Type VII secretion system” of diderm-mycolate (archetypal acid-fast) bacteria, i.e., the ESX. The T7SS is involved in the OM secretion and assembly of pili, which have been named very differently from one bacterial species to another (Sauer et al., 2004; Zav'yalov et al., 2010; Busch and Waksman, 2012; Thanassi et al., 2012), e.g., Afa/Dr (diffuse adherence fibrillar adhesin/Dr blood group antigen), AF/R1 (adhesive fimbriae on RDEC-1), AafD (aggregative adherence fimbriae), P pili, Type 3 fimbriae, Lpf (long polar fimbriae) or CS (coli surface) pili. More rationally, those pili can be categorized into (i) monoadhesive pili (represent the majority of cases), which display either thick rigid or thin flexible morphology and present a single adhesive domain at the tip, and (ii) polyadhesive pili, which display non-pilar, amorphous or capsule-like morphology and present two independent binding sites specific to different host-cell receptors for each of the subunits composing the organelle (Zav'yalov et al., 2010). Monoadhesive pili are also named FGS (F1-G1 short loop short) chaperone-assembled monoadhesin (e.g., Lpf) (Hung et al., 1996), and polyadhesive pili can either be FGL (F1-G1 long loop) chaperone-assembled polyadhesin (e.g., Afa/Dr) or FGS chaperone-assembled polyadhesin (e.g., AF/R1) (Zavialov et al., 2007). Of note, the so-called “alternate chaperone-usher pathway” (Soto and Hultgren, 1999) is obsolete and now included within the T7SS (Nuccio and Baumler, 2007; Poole et al., 2007; Zav'yalov et al., 2010; Thanassi et al., 2012). Pili secreted and assembled by the T7SS are involved bacterial adhesion, interbacterial interactions, aggregation, thereby promoting biofilm formation (Zav'yalov et al., 2010; Thanassi et al., 2012). Those pili can also initiate contact with host-cell receptors and mediate colonization of host cell surfaces. Interestingly, the adhesion of FimH pili (subfamily 2.5 FGS chaperone-assembled monoadhesins-5-1) is modulated by the shear force (Nilsson et al., 2006; Yakovenko et al., 2008; Le Trong et al., 2010).

### Pili assembled by the T8SS

The T8SS corresponds to the extracellular nucleation-precipitation (ENP) pathway (Desvaux et al., 2009b) involved in the OM secretion and assembly of thin and aggregative pili called curli (Hammar et al., 1996; Barnhart and Chapman, 2006; Dueholm et al., 2012; Hammer et al., 2012). Curli are functional amyloid fibers (Epstein and Chapman, 2008). They are involved in cell aggregation, bacterial adhesion and the formation of mature biofilms (Fronzes et al., 2008). They can actually change bacterial surface properties thereby enhancing adherence and attachment to surfaces. In the course of sessile development they constitute a significant part of the proteinaceous component of the biofilm matrix (Blanco et al., 2012). Curli also mediate host cell–bacteria interactions during infection (Wurpel et al., 2013).

### Adhesins secreted via the T9SS

The T9SS corresponds to the Por (porphyrin accumulation on the cell surface) secretion system (Sato et al., 2010, 2013; Shoji et al., 2011; McBride and Zhu, 2013). This secretion system has been essentially investigated in *Porphyromonas gingivalis* and *Flavobacterium johnsoniae* and seems restricted to members of the phylum *Bacteroidetes* (McBride and Zhu, 2013). Respective to surface colonization process, the T9SS is required for the secretion of cell-surface motility adhesins, namely SprB (colony-spreadingprotein B) and RemA (redundant motility protein A), but also some hemin-binding proteins (Nelson et al., 2007; Shoji et al., 2011; Shrivastava et al., 2012; Sato et al., 2013). The cell-surface adhesin SprB allows attachment to the substratum but is also required for efficient gliding as it is propelled along a closed helical loop track, generating rotation and translation of the bacterial cell (Nelson et al., 2007; Nakane et al., 2013a,b). While SprB is involved in movement over agar, RemA is involved in movement over surfaces coated with *F. johnsoniae* polysaccharide (Shrivastava et al., 2012). Gliding involves the rapid movement of the semiredundant motility adhesins SprB and RemA along the cell surface (Shrivastava et al., 2013). T9SS-secreted proteins exhibit C-terminal domains (CTDs) considered essential for attachment to the bacterial cell surface by an A-LPS anchor containing anionic polysaccharide repeating units (Kondo et al., 2010; Shoji et al., 2011; Slakeski et al., 2011; Shrivastava et al., 2013). CTD region function as a recognition signal for the T9SS and glycosylation occurs after removal of the CTD region (Shoji et al., 2011; Glew et al., 2012).

## Secreted proteins involved in surface colonization in monoderm bacteria

In monoderm bacteria, no protein secreted *via* the Tat, ABC exporter, holin, or Wss pathways has been reported as yet to be involved in bacterial adhesion and/or biofilm formation (Figure 3) but some proteins secreted by Sec, FPE, Tra and FEA have.

### Sec-secreted proteins involved in bacterial colonization

Once translocated by Sec, secreted proteins can have radically different locations in monoderm bacteria as they can either be (i) integrated or anchored to the CM, i.e., IMPs (inner membrane proteins) and lipoproteins, respectively, (ii) associated with the CW, i.e., parietal proteins (CW-proteins), (iii) subunits of pili, or (iv) released into the extracellular milieu and beyond (e.g., into a host cell) (Desvaux et al., 2006a, 2009b; Renier et al., 2012) (Figures 1, 3). Sec-secreted CW-proteins can either be anchored (i) covalently to the CW by a sortase if they exhibit an LPXTG domain (Ton-That et al., 2004; Schneewind and Missiakas, 2012), or (ii) non-covalently if they exhibit a CWBD (cell-wall binding domain), i.e., SLHD (S-layer homology domain), CWBD1 (CWBD of Type 1), CWBD2, LysM (lysin motif), WXL or GW motifs (Desvaux et al., 2006a). While no protein located at the CM has been reported to be involved in bacterial colonization in monodermata, several proteins located at the CW or forming cell-surface supramolecular complexes were reported to participate to this process.

#### Colonization factors located at the cell wall

In monoderm bacteria, proteins of the Bap family exhibit a C-terminal LPXTG domain enabling their covalent anchoring to the CW by sortases (Lasa and Penadés, 2006; Latasa et al., 2006). While Bap is involved in biofilm formation in *S. aureus* (Cucarella et al., 2001), the *bap* gene has never been found in *S. aureus* human isolates but only in isolates associated with ruminant mastitis (Lasa and Penadés, 2006). Strains encoding *bap* show lower adherence to some ECM proteins (fibrinogen and fibronectin) but also epithelial cell cultures, suggesting Bap might act as an anti-attachment factor preventing initial attachment to host tissues (Cucarella et al., 2002). At the same time, Bap facilitates the colonization of host tissues and the establishment of persistent infections by *S. aureus* (Cucarella et al., 2004; Valle et al., 2012). The interaction of Bap with Gp96/GRP94/Hsp90 provokes a significant reduction of epithelial cell invasion by interfering with the fibronectin binding protein invasion pathway. While Bap is often considered as a key determinant for biofilm formation (Lasa, 2006), a recent investigation revealed that there is no direct correlation with biofilm formation and that a single gene or subset of genes cannot be utilized as a biofilm indicator for morphology in *S. aureus* (Tang et al., 2013). Interestingly, Bap is subjected to phase variation and the switch between Bap ON/Bap OFF states might regulate sessile development (Henderson et al., 1999; Tormo et al., 2007). In *Enterococcus faecalis*, Esp (enterococcal surface protein) also leads to a significant increase in biofilm formation (Tendolkar et al., 2004) and contributes to persistence in the host (Sava et al., 2010). In *S. epidermidis* and *Listeria monocytogenes*, however, Bhp (Bap homologue protein) and BapL, respectively, are not clearly involved in biofilm formation (Tormo et al., 2005; Lasa and Penadés, 2006; Jordan et al., 2008; Renier et al., 2011).

As recently reviewed (Chagnot et al., 2012), different MSCRAMM surface proteins have been investigated in monoderm bacteria (Vengadesan and Narayana, 2011). Among the main ECM fibrillar proteins, namely collagen, fibronectin, laminin and elastin, eight fibronectin-binding domains (FBD1 to FBD8) and only one collagen-binding domain (CBD) have been characterized to date and much remains to be learned about specific binding to other ECM components (Chagnot et al., 2012). Very interestingly, domains involved in binding to ECM proteins such as fibronectin and collagen could also bind polystyrene as demonstrated with RspA (*Rhusiopathiae* surface protein A) (Shimoji et al., 2003).

In *B. subtilis*, the S-layer protein BslA (*Bacillus* S-layer protein A; formerly YuaB) is important for pellicle biofilms (Ostrowski et al., 2011; Vlamakis et al., 2013). BslA has amphiphilic properties and forms a hydrophobic layer on the surface of the biofilm which can contribute to colonization of the air–surface interface (Kovacs and Kuipers, 2011; Kobayashi and Iwano, 2012).

#### Pili: type 3 (T3P) and amyloid fibers

A little bit more than a decade ago, pili were considered to be absent from monoderm bacteria. The first pathway recognized in pilus biogenesis in monodermata involves polymerization of LPXTG-pilins by different types of transpeptidase sortases, (Shimoji et al., 2003; Ton-That and Schneewind, 2003; Ton-That et al., 2004; Telford et al., 2006; Kang and Baker, 2012). Those pili of Type 3 (T3P) are involved in adhesion, biofilm formation and host colonization (Swierczynski and Ton-That, 2006; Danne and Dramsi, 2012). LipA (light-inducible pilin A) from *Arthrobacter photogonimos* was proposed as the major pilin of an alternative pilus formation pathway in monoderm bacteria (Yang and Hoober, 1998, 1999; Ton-That and Schneewind, 2004; Swierczynski and Ton-That, 2006).

In *B. subtilis*, pili formation of the amyloid-fiber type has been recently demonstrated to be essential in colony and pellicle biofilms but not for surface-adhered biofilms (Romero et al., 2010; Vlamakis et al., 2013). The CW-protein TapA (TasA anchoring and assembly protein A; formerly YqxM) allows the CW anchoring and assembly of TasA (translocation-dependent antimicrobial spore component A) into long amyloid fibers (Stover and Driks, 1999; Romero et al., 2011), these proteins are not required in biofilms. In *Peptostreptococcus micros*, auto-aggregative pili are formed at least from a CWBD1 protein called FibA (fibril-like structure subunit A) (Kremer et al., 1999).

#### Cellulosome

Cellulosomes are supramolecular protein complexes found on the cell surface of some cellulolytic anaerobic monoderm bacteria, such as the thermophilic *Clostridium thermocellum* or mesophilic *C. cellulolyticum*, and dedicated to specific adhesion, colonization and degradation of insoluble lignocellulosic substrates (Bayer et al., 2004, 2008; Gilbert, 2007; Fontes and Gilbert, 2010). It is also responsible for regulating the entering carbon metabolic flux in the bacterial cell, which in turn can influence the bacterial colonization of the insoluble lignocellulosic substratum (Guedon et al., 1999, 2000; Payot et al., 1999; Desvaux et al., 2001; Desvaux and Petitdemange, 2001, 2002; Desvaux, 2004). The cellulosome is composed of a non-catalytic protein called scaffoldin, which exhibits domains called cohesin of Type I (Coh I) domains allowing the specific binding of a dockerin domain of Type I (Doc) borne by cellulosomal enzymes. Actually, a single Doc I can simultaneously bind two Coh I and thus allow the linking of two scaffoldins resulting in the formation of polycellulosomes (Carvalho et al., 2003). In *C. thermocellum*, a cellulosome has a size ranging from 2.0 to 6.5 MDa depending on the bacterial strain (Béguin and Lemaire, 1996) and forms polycellulosomal protuberances of up to 100 MDa (Shoham et al., 1999). The cellulosomal enzymes and/or the scaffoldin contain carbohydrate-binding modules (CBMs) involved in the tight binding to a carbohydrate-polymer such as cellulose (Shoseyov et al., 2006; Guillen et al., 2010). The scaffoldin can also exhibit a Doc II domain that can interact with Coh II present in SLHD cell-wall proteins, thus permitting cell-surface display of the cellulosome. In *C. cellulolyticum* for instance, the molecular mechanisms for attachment of the cellulosome to the bacterial cell surface remains unknown (Desvaux, 2005a,b). The architecture of the cellulosome can be even more complex with the assembly of scaffoldins on other scaffoldins like in *Acetivibrio cellulolyticus* where one supramolecular complex can potentially bind 84 different cellulosomal enzymes (Rincon et al., 2003; Xu et al., 2003, 2004). However, the molecular mechanisms permitting the coordinated assembly of the different protein subunits on the bacterial cell-surface remain unclear (Desvaux, 2005b, 2006).

### Type 4 pilus (T4P) assembled by the FPE

The Com (Competence development) pathway involves both the bacterial competence-related DNA transformation transporter and the FPE system (Dubnau, 1997, 1999; Dubnau and Provvedi, 2000; Chen and Dubnau, 2004). In *B. subtilis*, the FPE system is involved in the formation of a pseudopilus from Type 4 prepilins (Chen and Dubnau, 2004; Chen et al., 2006) but do not form a complete Type 4 pilus (T4P). While this was considered as a paradigm for monoderm bacteria, it was recently demonstrated the FPE could form complete Type 4 pilus in *Streptococcus pneumonia* (Laurenceau et al., 2013). While this T4P was only considered in the context of DNA transformation, its implication in bacterial colonization as reported for T4P in diderm-LPS bacteria is an intriguing possibility that would require further investigations in other species such as *L. monocytogenes* (Rabinovich et al., 2012). GP25 (glycoprotein of 25kDa) from *Ruminococcus albus* was characterized as the major protein subunit of T4P present at the bacterial cell surface (Pegden et al., 1998; Mosoni and Gaillard-Martinie, 2001; Rakotoarivonina et al., 2002, 2005). Those pili could be involved in cellulose colonization and cellulose degradation. T4P involved in biofilm formation and gliding motility was also uncovered in *Clostridium perfringens* (Varga et al., 2006; Mendez et al., 2008; Varga et al., 2008).

Protein components of the FPE machinery are homologous to some proteins found in T2SS, T4SS, and T4P assembly apparatus in diderm-LPS bacteria and have been collectively called PSTC (Pilus/Secretion/Twitching motility/Competence) (Fussenegger et al., 1997; Dubnau, 1999; Peabody et al., 2003). In *Bifidobacterium breve*, a functional Tad system involved in the formation of T4bP has recently been uncovered (O'connell Motherway et al., 2011). These pili are essential for intestinal colonization of the host. Besides the genus *Actinobacteria*, a Tad system was also identified in *Clostridia*, namely *Cl. acetobutylicum* (Desvaux et al., 2005b). Again, there is much confusion between the pili assembly by FPE, Tad and the misleading called “Type IV-like secretion system” in monoderm bacteria corresponding more adequately to the Tra system. This would necessitate in-depth phylogenetic and functional-genetic analyses to clarify the situation.

### Flagellum assembled by the FEA

In monoderm bacteria, the flagellar subunits are secreted and assembled by the FEA but the involvement and exact contribution of flagella in biofilm formation remain difficult to establish. Taking *L. monocytogenes* as a case study, it is clear that the regulation of flagella expression is quite complex with the intervention of several transcription regulators but basically that flagella are not expressed at temperatures higher than 30°C and bacterial cells are then non-motile (Renier et al., 2011). Regarding listerial colonization, it was shown flagella were not essential for biofilm development but facilitated initial attachment to surfaces (Vatanyoopaisarn et al., 2000) and were even crucial for initial adhesion (Tresse et al., 2006). During infection, though, the flagella would act as mediators of motility rather than adhesins to enhance the adhesion of *L. monocytogenes* to targeted host cells (O'neil and Marquis, 2006). By contrast with early findings, it was further shown the role of the flagella as mediators of motility rather than adhesins was critical for initial surface attachment and subsequent biofilm formation (Lemon et al., 2007). In dynamic culture conditions for sessile development (i.e., using flow cells), the loss of the flagella resulted in the formation of a very dense biofilm (hyperbiofilm) (Todhanakasem and Young, 2008). These conflicting findings could arise from the use of different *L. monocytogenes* strains as some of them can be more or less laboratory-adapted (domesticated) and have different regulations of flagella expression (Grundling et al., 2004). It could also result from the use of different culture conditions, such as the growth media (e.g., complex undefined or chemically defined), temperature (e.g., related to infection or environmental conditions), support (e.g., plasticware, stainless steel or glass) and/or culture systems (e.g., static or dynamic conditions) (Renier et al., 2011).

In *Bacillus cereus*, flagella motility (i) is necessary for biofilm formation at air-liquid interface as it allows bacteria to reach it, (ii) promotes recruitment of planktonic cells within the biofilm by allowing motile bacteria to invade the whole biofilm, and (iii) allows spreading of the biofilm on surfaces (Houry et al., 2010). In *B. subtilis*, however, motility and biofilm formation are quite clearly coordinately and oppositely controlled (Kearns et al., 2005; Newman et al., 2013; Vlamakis et al., 2013). It was further demonstrated a protein clutch arrested flagellar motilily by disengaging motor force-generating elements in cells engaged in sessile development (Blair et al., 2008). Nonetheless, and while the number of motile cells decreases in the course of sessile development, a subpopulation of motile cells remains in mature biofilms (Vlamakis et al., 2008) and a biofilm exclusively composed of unflagellated cells is delayed in forming pellicle biofilms (Kobayashi, 2007) and defective in the formation of surface-adhered biofilms (Chagneau and Saier, 2004). Interestingly, motile *Bacillus thuringiensis* can tunnel deep within a biofilm structure (Houry et al., 2012). Those swimming cells create transient pores that increase irrigation within the biofilm. This can either improve biofilm bacterial fitness by increasing nutrient flow in the matrix or facilitating the penetration of toxic substances detrimental for biofilm formation.

## Non-classical protein secretion and bacterial colonization

Analysis of the protein content of the extracellular milieu and/or the cell surface, revealed the presence of unexpected proteins primarily predicted or known to be localized in the cytoplasm, with no N-terminal signal peptide (SP), and no dedicated protein secretion system. While early investigations suggested it could result from bacterial cell lysis, it later appeared some of those proteins were actually secreted by previously unrecognized pathways, e.g., some SecA2-dependent secreted proteins (Desvaux and Hébraud, 2006, 2009; Rigel and Braunstein, 2008; Renier et al., 2013). Non-classical (NC) protein secretion refers to the uncharacterized mechanisms responsible for secretion of those proteins (prior to characterization such apparent secretion could be the result of phenomenon unrelated to secretion *per se*, such as membrane budding, allolysis, phage-mediated lysis, GTA, etc…) (Bendtsen et al., 2005; Bendtsen and Wooldridge, 2009). Another unexpected finding was the involvement of some of those proteins in an additional function completely different form the primary one. Indeed, the glyceraldehyde-3-phosphate dehydrogenase (GAPDH) is a well-known and key glycolytic enzyme but when present on the bacterial cell surface it exhibits very significant binding activity against plasmin(ogen), fibrinogen, and human cells (C1q component of the classical component pathway) (Bergmann et al., 2004; Jin et al., 2005; Egea et al., 2007; Matta et al., 2010; Tunio et al., 2010; Terrasse et al., 2012). Several of those so-called moonlighting proteins have been identified in bacteria and play a significant role in bacterial colonization, especially in an infection context (Jeffery, 2003, 2009; Huberts and Van Der Klei, 2010; Copley, 2012; Henderson and Martin, 2013). Most enzymes of the EMP (Embden-Meyerhof-Parnas) pathway can moonlight and a role as an adhesin is quite common, e.g., for enolase, aldolase, or phosphoglucomutase (PGM) (Henderson and Martin, 2011, 2013). However, the molecular mechanisms responsible for their bacterial cell-surface localization remain unknown.

## Conclusions and perspectives

While extremely useful, the secretome concept has not been fully exploited and is still in its infancy. In the context of bacterial colonization, it allows a rational approach for comprehending the secreted proteins directly involved in bacterial adhesion and/or biofilm formation. Considering the protein routing and secretion mechanisms embrace the idea that some of those adhesins and/or adhesion supramolecular complexes share common post-translational and post-translocational maturation mechanisms that may lead to the identification of novel targets for therapeutic strategies. While the majority of investigations are focused on colonization factors in the context of infection, their involvement in the interactions of commensal bacterial, as well as bacteria in an environmental context, are also important areas and should not be overlooked. Bacterial colonization is not the prerogative of pathogens and there is much to gain from the study of colonization of host species by commensal bacteria, as well as environmentally orientated studies. Clearly, there are numerous proteins and supramolecular structures involved in bacterial adhesion and biofilm formation but their functional characterization still demands extensive investigations, e.g., alternative pili in monoderm bacteria or collagen-/laminin-/elastin-binding domains. The understanding of the global and coordinated regulation of the secretome in the course of colonization at the single cell, homogenous population, and multi-species microbial community levels is a major challenge in different ecological niches. Phase variation for instance is a well-known phenomenon especially for factors involved in colonization process but is rarely considered and is poorly understood at a global scale. In the last couple of years, new secretion system such as the T6SS or T9SS have been unexpectedly uncovered and there is much to bet that other protein secretion system(s) are yet to be discovered. Besides, the exact contribution of NC secretion and other protein trafficking mechanisms in bacterial colonization (e.g., allolysis, phage-mediated lysis, membrane budding or GTA) remains to be determined. Regarding the different secretion systems present in a single bacterial cell and numerous systems within microbiota as well as their associated secreted proteins involved in colonization, they promise to be the subject of a lot of research work ahead as well as exciting new developments in the field.

### Conflict of interest statement

The authors declare that the research was conducted in the absence of any commercial or financial relationships that could be construed as a potential conflict of interest.
